# Intraoperative graft-related complications are a risk factor for recurrence in arthroscopic Latarjet stabilisation

**DOI:** 10.1007/s00167-019-05400-x

**Published:** 2019-02-22

**Authors:** Bartłomiej Kordasiewicz, Konrad Małachowski, Maciej Kiciński, Sławomir Chaberek, Andrzej Boszczyk, Dariusz Marczak, Stanisław Pomianowski

**Affiliations:** 1 Trauma and Orthopaedics Department, SPSK im. A. Grucy w Otwocku, ul. Konarskiego 13, 05-400 Otwock, Poland; 2 Centre of Postgraduate Medical Education, Warsaw, Poland; 3 Department of Orthopaedics, Otwock, Poland

**Keywords:** Anterior shoulder instability, Coracoid bone block, Latarjet stabilisation, Arthroscopy, Glenoid bone loss

## Abstract

**Purpose:**

The goal of this study was to evaluate clinical and radiological outcomes after arthroscopic Latarjet stabilisation in anterior shoulder instability.

**Methods:**

Ninety-three patients after primary arthroscopic Latarjet stabilisation were reviewed. Satisfaction, subjective shoulder value (SSV), Walch–Duplay and Rowe scores, and range of motion and stability were evaluated on clinical examination. Computed tomography (CT) was used to analyse graft position and fusion.

**Results:**

Ninety patients (96.8%) were available for clinical and 85 for CT evaluation. The mean follow-up was 23.7 months (13–50, SD 7.1) and age at surgery was 26.2 years (16–44, SD 5.6). Intraoperative complications were reported in eight patients (8.9%) and recurrence in three (3.3%). Significantly, two out of three patients with recurrence had intraoperative graft complications (*p* = 0.0107). Forty-one patients (45.6%) reported the feeling of “subjective return to sport anxiety”. External rotation with arm at the side was 59° (10–90°, SD 20) with 15° (0–70°, SD 17) of loss of rotation. These two factors correlated with results the most. Patient satisfaction was evaluated as 92% (40–100, SD 14) and SSV 90% (30–100, SD12). Revision rate after primary surgery was 10%. CT showed graft healing in 81 (95.3%) patients. A graft position between 2 and 5 o’clock was found in 70 (83.4%) patients and flush to the anterior glenoid rim in 34 (40.5%). Osteolysis of the superior part of the graft was found in 55 (64.7%) patients. CT evaluation showed no correlation with clinical results.

**Conclusion:**

Arthroscopic Latarjet stabilisation demonstrates satisfactory results in short-term follow-up; however, intraoperative graft-related complications are a risk factor for recurrence. “Subjective return to sport anxiety” and loss of external rotation with the arm at the side are factors worsening the results. Graft position imperfections and osteolysis of the superior part of the graft reported in CT evaluation do not influence the clinical results.

## Introduction

Latarjet coracoid bone block stabilisation is one of the most efficient surgical procedures in anterior shoulder instability [1–6]. The open technique remains the “gold standard”; however, the number of arthroscopic stabilisations conducted has been increasing [7–16]. The arthroscopic technique might need some improvement and modification to become the preferred method of treatment, so proper identification of limitations is mandatory. Current literature offers only a limited number of studies, usually with small patient numbers, making this evaluation difficult [8, 11, 12, 14, 16–22]. This study presents both clinical and computed tomography (CT) outcomes of a large series of patients operated on by a single surgeon. It was hypothesised that evaluation of this large cohort of patients allows identification of surgical and radiological factors influencing the results and increasing the risk of complications and recurrence.

## Materials and methods

Two-hundred and sixteen patients with anterior shoulder instability were operated on by the first author from 2011 to 2016 in the Trauma and Orthopaedics Department*. In this group, 104 (48.1%) arthroscopic Latarjet stabilisations were performed. Based on radiological (routine AP and Y radiographs, supplemented with CT or MRI) and clinical data, the indications for soft tissue or bone block procedure were made preoperatively. Patients indicated for Latarjet stabilisation were supposed to have several risk factors: professional sport or high-risk activity, Hill–Sachs lesion of more than 15% of humeral head diameter, glenoid bone loss > 10%, laxity (thumb–forearm distance less than 2 cm, external rotation with arm at the side > 85°), and recurrence after a prior soft tissue procedure. The final operative decision was undertaken after arthroscopic glenohumeral joint inspection encompassing anterior soft tissue quality (poor tissue quality in favour of the Latarjet procedure) and assessment of Hill–Sachs lesion engagement according to the “on-track off-track” hypothesis [23, 24].

### Surgical technique

Arthroscopic stabilisation was performed according to Lafosse’s technique, using specific arthroscopic instruments (DePuy, Mitek, Raynham, MA, USA) in the beach chair position under general anaesthesia and interscalene block [15]. The first step in the procedure was confirmation of proper indication and associated soft tissue injury repair. Then, the rotator cuff interval was opened and the anterior capsule removed. The scope was switched into the antero-lateral portal and a “switching stick” was inserted from the posterior portal into the subscapularis muscle (inside-out technique) to mark the subscapularis split level. In the next step, a subscapularis split was performed using a radiofrequency probe at the previously marked level and the anterior glenoid neck was prepared using a burr. Next, preparation around the coracoid began: the pectoralis minor and coraco-acromial ligament were released and the coracoid ventral surface was gently decorticated. Two holes were drilled and washers (“top-hats”) were placed in the drilled coracoid. The coracoid was cut off and attached to the plastic handle introduced through the pectoralis major muscle (medial portal). The final step was bone block positioning at the anterior glenoid rim and fixation with two cannulated screws. Postoperatively, a simple sling was used for 2–10 days for pain control. Passive pendulum shoulder exercises were introduced immediately after surgery. After pain reduction, the sling was discontinued and active shoulder exercises were started. Gentle stretching exercises were introduced in the third week and after achieving full forward flexion, muscle-strengthening exercises were initiated, no sooner than 8 weeks after surgery. Contact sports were allowed after restoration of a full range of motion and strengthening, but no sooner than 3 months after surgery.

### Patient evaluation

**Fig. 1:**
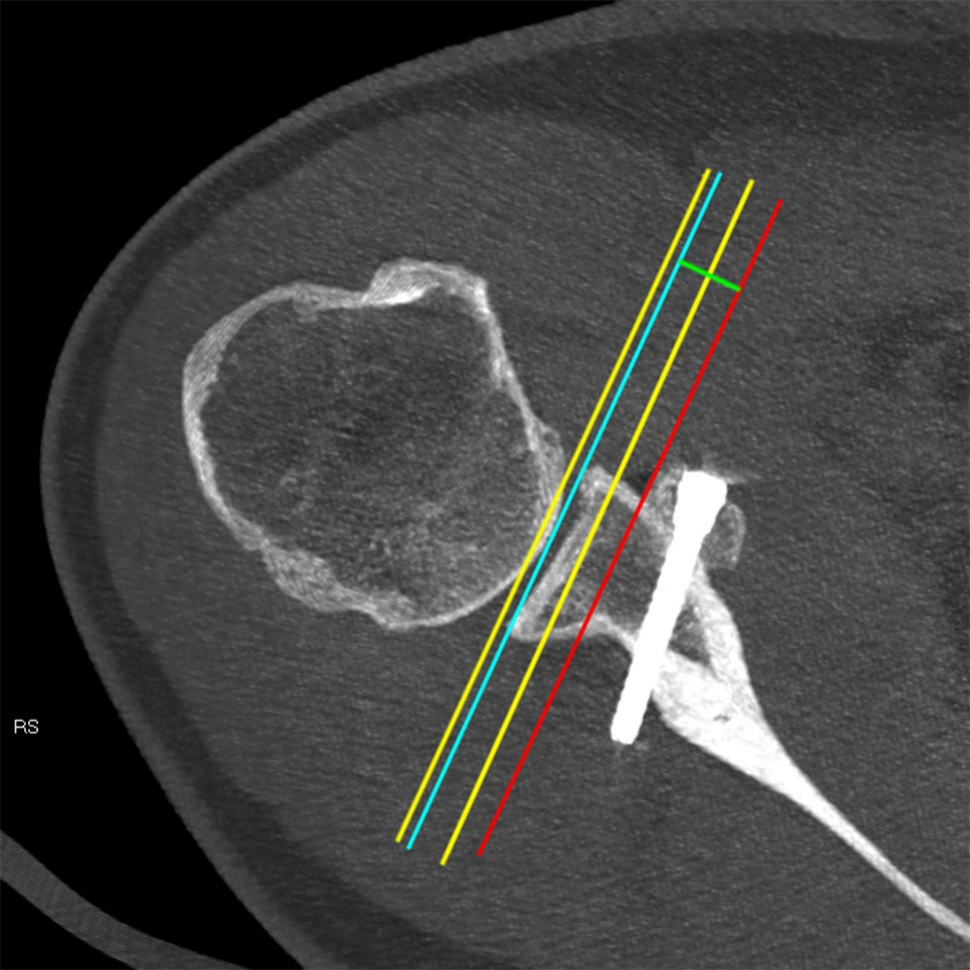
Axial view—bone block healed in a medial position. The blue line indicates the line of reference between the anterior and posterior glenoid rim; the yellow lines are medial (4 mm) and lateral (2 mm) tolerance lines—the zone of “tolerance” is between these yellow lines; the red line indicates the most medial point of the lateral border of the graft; the green line shows the distance between the reference line and the lateral border of the graft

**Fig. 2:**
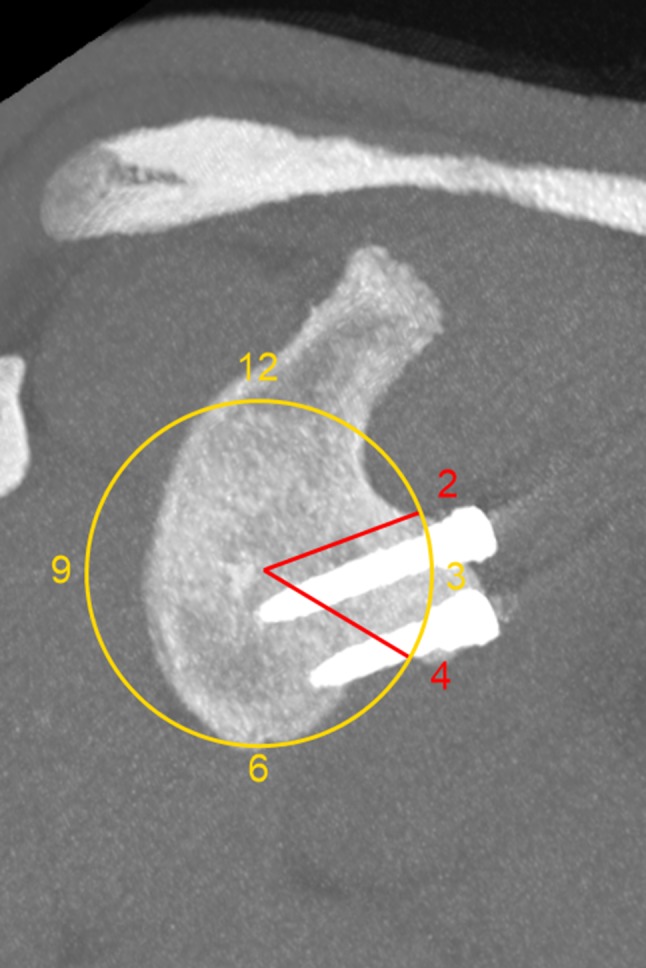
Saggital view—bone block healed between 2 and 4 o’clock

**Fig. 3:**
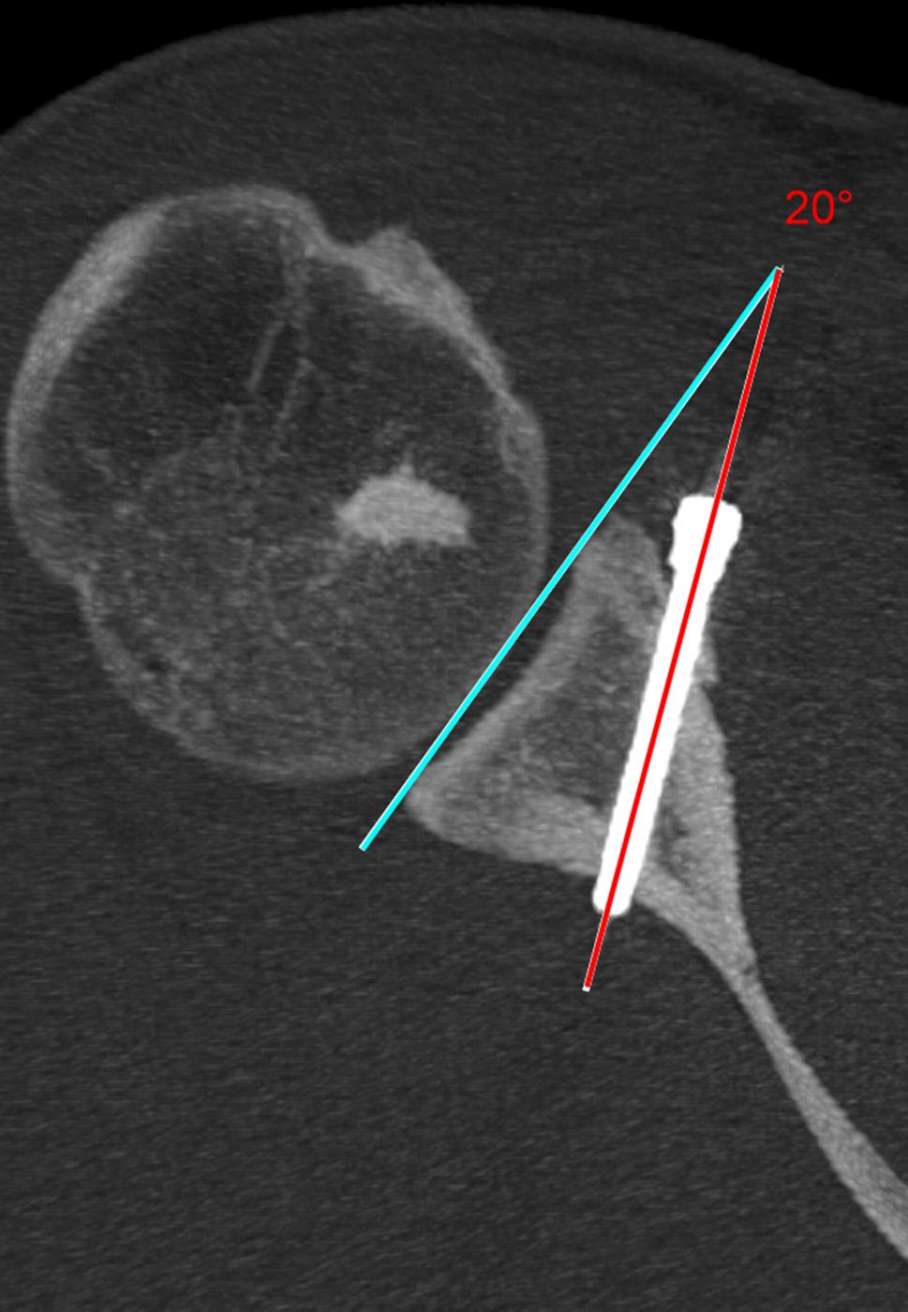
Axial view—the screw angle. The angle created between the reference line (blue line) and the axis of the screw (yellow line). Partial osteolysis of the graft at the level of the screw is visible

**Fig. 4:**
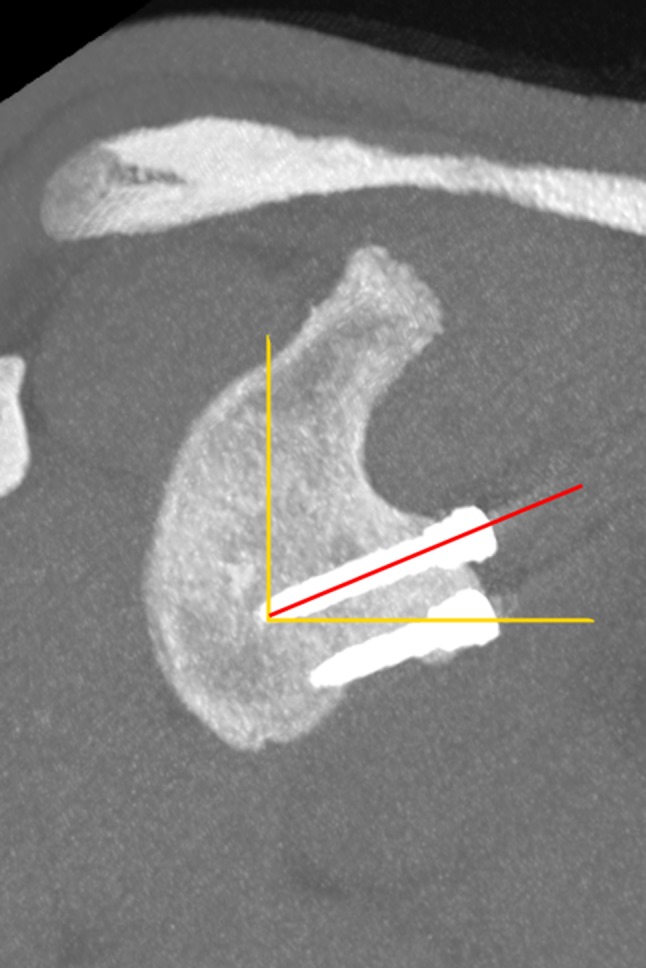
Saggital view—screw-equator angle. The angle measured between the equatorial line (yellow horizontal line perpendicular to yellow vertical line between superior and inferior glenoid pole—12 and 6 o’clock) and the axis of the screw (red line). Cronbach’s α for the screw-equator angle (0.61 for intra-rater and 0.77 for inter-rater) implies this measurement is unreliable. Due to the complex shape of the glenoid and alterations of the anterior glenoid rim in shoulder instability, it is difficult to achieve the exact same image for repeated measurements. Despite this fact, no correlation was found between this parameter and other clinical and radiological results. To our knowledge, this parameter value was not yet reported in the literature

Ninety-three patients after primary arthroscopic Latarjet stabilisation were followed up clinically and with CT evaluation at a minimum of 13 months. All patients after revision Latarjet stabilisation were excluded from the study. Informed consent was obtained from all the individuals included in the evaluation. This study achieved institutional review board approval (Ethical Board of the Centre of Postgraduate Medical Education, Warsaw, Poland, ID 38/PB/2014). Clinical and radiological evaluations were performed by two senior residents. Clinical results were assessed with Walch–Duplay, Rowe and simple shoulder value (SSV) scores and pain via visual analog scale (VAS) [25–28]. Patients also evaluated satisfaction by answering the question (rating from 0 to 100%): “How satisfied are you with the surgery outcome?” CT scans were performed on a GE Bright Speed 16-row scanner, using the standard shoulder protocol and slice thickness 0.63 mm. All the measurements were made using Carestream software version 11.4 (Carestream Health; Rochester, NY, USA). Three dimension (3D) and multiplanar reformations were used for optimal visualisation of the anatomy and screws. Graft fusion was determined by the presence of a bone bridge between the coracoid and the glenoid. Non-unions were identified as stable—with no lysis around the screws, and unstable—with hardware loosening and graft dissociation. Bone block osteolysis was evaluated in both the axial and sagittal planes and described as total—concerning the entire graft, or partial—around the superior or the inferior screw only. The bone block position was evaluated according to Kraus et al. technique [29]. In the axial view, the line between the anterior and posterior glenoid rim served as the reference line. The graft could be positioned flush, medial or lateral to this glenoid line—a perpendicular line between the reference line and the lateral border of the graft was measured in millimetres. The graft was considered as “flush” if the entire graft was in this position or osteolysis at the superior part of the graft which was associated with the flush position of the lower part—partial osteolysis of the graft was already reported [30, 31]. The graft was considered as “medial” if the entire graft was medial to the reference line and the distance between the most medial point of the lateral border of the graft and the reference line was measured (Fig. 1). If any part of the graft was lateral to the reference line—the distance was measured and graft was considered as “lateral”. Some authors have assumed that the acceptable graft translation is 4–5 mm medially to the glenoid rim [11, 32]. It is also important that the glenoid cartilage remains the reference point during surgery. Zumstein et al. assessed glenoid cartilage thickness to be about 2.3 mm [33]. For these reasons, an appropriate graft placement could be judged as between 4 mm medially and 2 mm laterally—these two border values were also evaluated in this study (Fig. 1). The bone block position in the vertical axis, i.e., graft height (Fig. 2), was evaluated in the sagittal plane using the “clock system” [29]. The axis connecting the most superior and the most inferior aspect of the glenoid formed a vertical line between 12 and 6 o’clock points. The anterior glenoid was always considered between the 12 and 6 o’clock points (with 3 o’clock always anteriorly in half distance) and was divided into four sectors (1–3, 2–4, 3–5 and 4–6 o’clock position). Screw orientation in relation to the glenoid was measured in the axial plane as proposed by Ladermann et al.: screw angle was determined as the angle between the line linking the posterior and anterior glenoid rim and the screw axis (Fig. 3) [34]. In the same plane, screw protrusion in relation to the posterior glenoid neck cortex was measured to evaluate its penetration into the infraspinatus fossa. Screw-equator angle was measured between the line perpendicular to the glenoid meridian and the screw axis in the sagittal plane at the same level as graft height was evaluated (Fig. 4). All the measurements were rounded to one decimal place. The two measurements were performed by two independent investigators for each parameter. For intra-rater variability assessment, the measurements were repeated at 2-week intervals. The subscapularis muscle fatty infiltration was evaluated according to Goutallier et al. classification [35].

### Statistical analysis

Statistical analysis was performed using STATISTICA 7.0 software (StatSoft, Inc., STATISTICA for Windows, Tulsa, OK). Descriptive data were presented as mean, range (min/max), median, standard deviation (SD) and % values. Correlations between the parameters were calculated with Spearman Rank Order coefficient significant at *p* < 0.05.

Post hoc power analysis for preoperative and postoperative Rowe and Walch–Duplay scores, 90 patient group and *α* error rate 0.05, was evaluated as 1.000 (100%). Post hoc sample size was calculated for the same preoperative and postoperative scores—assuming *α* error rate of 0.05 and power of 95%—four patients in the group should be included to reject the null hypothesis. For CT measurements, inter-rater and intra-rater reliability was tested by calculating Cronbach’s α for each measurement repeated by a single investigator and between the two investigators.

## Results

Ninety patients were available for clinical evaluation (96.8%)—three patients were lost to follow-up. Eighty-five patients were available for complete CT evaluation. The mean follow-up was 23.7 months (SD 7.1) and age at surgery was 26.2 years (SD 5.6). Detailed patient characteristics are reported in Table 1.

**Table 1:** Patient preoperative data

	Number of patients
Patients available for f-up	90
Sex F/M	10 (11.1%)/80 (88.9%)
Dominant arm	55 (61.1%)
Pain during live activity	22 (24.4%)
Laxity	55 (61.1%)

	Mean value	Median	Range values	SD
Age at first episode (years)	21.2	20	13–40	5.2
Age at surgery (years)	26.2	26	16–44	5.6
Follow-up (months)	23.7	24	13–50	7.1
Time to surgery (months)	59	46	4–228	47.6
Number of dislocations	4	3	0–40	7
Number of subluxations	13	5	0–100	20
Walch–Duplay score	21	25	− 10 to 40	9
Rowe score	27	30	0–40	6

In brackets % values

SD—standard deviation

### Intraoperative data

The mean time of surgery was 113 min (median 110, range 70–210, SD 27). Concomitant injuries were identified and repaired in nine (10%) patients (Table 2). Hill–Sachs lesions were found in all 90 patients. Eight (8.9%) intraoperative complications were reported (Table 2). Seven out of eight intraoperative complications concerned graft harvesting, drilling or fixation. Correlation was found (*p* = 0.0107) between intraoperative complications and recurrence: two patients with intraoperative problems (graft breakage with one screw fixation and destruction of the peripheral cortex whilst drilling) had recurrence. The remaining six complications had no impact on the results.

**Table 2:** Intraoperative data

	Number of patients	Type of lesions and complications
Concomitant injuries	9 (10%)	4 SLAP lesions 1 partial supraspinatus tear 1 SLAP lesion with loose bodies 1 loose body 1 posterior labrum tear 1 isolated LHB tendon tear 73 HS type I (81.1%) 17 HS type II (18.9%)
Intraoperative complications	8 (8.9%)	1 medial cutaneous antebrachial nerve injury 1 graft breakage at the proximal hole level, fixed with 1 screw 2 graft ventral side infractions without any influence on final fixation 1 superior screw fixed too deep in the graft due to poor bone quality* 2 distal cortices destroyed whilst drilling a distal hole in the graft* 1 distal screw poor fixation*

In brackets % values; HS—Hill–Sachs lesion, type I—in proximity of infraspinatus tendon insertion, type II—located more medially and separated from infraspinatus tendon insertion by the cartilage insula; *in these patients no compression was achieved with only anti-rotational effect of the second screw

### Clinical results

Significant improvement (*p* = 0.00) was reported in Walch–Duplay score from mean 21 (SD 9) to 79 (SD 19) and Rowe score from mean 27 (SD 6) to 81 (SD 19). Patient satisfaction was evaluated as mean 92% (SD 14) and SSV as 90% (SD 12)—Table 3. The mean forward flexion and abduction was 177° (SD 12 and 13, respectively). External rotation with arm at the side was 59° (SD 20) with 15° (SD 17) of loss of rotation compared to the contralateral shoulder. Significant correlations were found between these three parameters and Walch–Duplay, Rowe and SSV scores, with the strongest influence of loss of external rotation with arm at the side (Table 4). Recurrence was reported in three (3.3%) patients—one dislocation and two subluxations. In all these patients, three screw fractures and two postoperative graft fractures were found (correlation of postoperative screw and graft fracture with recurrence, *p* = 0.00). As mentioned above, two out of three cases of recurrence had intraoperative complications. All the patients with recurrence were revised (Table 5). Forty-one patients (45.6%) reported the feeling of “subjective return to sport anxiety” (SRSA)—the term denoting a patient’s incertitude about returning to overhead activity, which was neither confirmed on clinical examination nor in the patient’s satisfaction survey. However, this factor had a strong influence (correlation − 0.634 for Walch–Duplay and − 0.758 for Rowe scale, *p* < 0.05) on the results: patients with SRSA received a mean 66 points (SD 20) in Walch–Duplay and 67 points (SD 18) in Rowe scores, whereas patients without SRSA received 89 (SD 11) and 93 points (SD 8) respectively. Eight revisions were performed after the initial surgery and one patient was still hesitating at the moment of this publication writing (Table 5). If this potential patient was added, the number of revisions after primary arthroscopic Latarjet stabilisation would increase to 9 (10% reported in Tables 3, 5).

**Table 3:** Postoperative results

	Mean value	Median	Range values	SD
Walch–Duplay score	79	80	0–100	19
Rowe score	81	82	15–100	19
Satisfaction (%)	92	100	40–100	14
SSV (%)	90	90	30–100	12
Flexion (°)	177	180	70–180	12
Abduction (°)	177	180	70–180	13
ER1 (°)	59	60	10–90	20
Delta ER1 (°)	15	10	0–70	17
ER2 (°)	82	85	30–95	10
Delta ER2 (°)	6	5	0–60	9
VAS	1	0	0–8	2

	Number of patients
Subjective return to sport anxiety	41 (45.6%)
Recurrence	3 (3.3%)
Revision	9 (10%)

In brackets % values

*SSV* simple shoulder value, *ER1* external rotation with arm at the side, *Delta ER1* loss of rotation with arm at the side compared to the contralateral shoulder, *ER2* external rotation with arm in 90° of abduction, *Delta ER2* loss of external rotation with arm in 90° of abduction comparing to the contralateral shoulder, *VAS* visual analog scale, *SD* standard deviation

**Table 4:** Correlations between flexion, abduction, loss of external rotation with arm at the side and clinical scores

	Walch–Duplay	Rowe	SSV
Flexion	*R* = 0.4 *p* = 0.00005	*R* = 0.4 *p* = 0.00013	*R* = 0.3 *p* = 0.00906
Abduction	*R* = 0.3 *p* = 0.0003	*R* = 0.4 *p* = 0.00017	*R* = 0.3 *p* = 0.0048
Delta ER1	*R* = − 0.6 *p* = 0.0	*R* = − 0.5 *p* = 0.0	*R* = − 0.4 *p* = 0.0

Data evaluated by Spearman Rank Order Correlation test, *R* = strength of correlation; statistically significant when *p* < 0.05 (in this table only significant values are presented)

*SSV* simple shoulder value

**Table 5:** Details of revisions after initial primary arthroscopic Latarjet stabilisation

Number of patients	Problem	Type of revision surgery
3	Recurrence	2 iliac crest bone grafts 1 remplissage procedure (graft healed and intact)
2	Graft osteolysis and screw loosening	Screw removal
1	Frozen shoulder	Arthroscopic arthrolysis
1	Posterior labrum injury—new trauma	Posterior labrum repair
2	Discomfort in the infraspinatus area—screw protrusion	1 screw removal 1 patient is hesitating (potentially screw removal)

### CT measurement reliability

Cronbach’s *α* for intra-rater reliability was 0.88, 1.0, 0.98 and 0.61 for graft position medial to lateral, graft position in the o’clock system, screw angle and screw-equator angle. Respectively, for inter-rater reliability these values were 0.89, 0.82, 0.96 and 0.77.

### Computed tomography evaluation

Graft fusion was reported in 81 patients (95.3%), total graft osteolysis in 1 (1.2%), stable pseudoarthrosis as well as graft fracture in 2 (2.3%)—Table 6. Graft osteolysis at the level of the superior screw was found in 55 patients (64.7%), as graft osteolysis at the level of the inferior screw—in 2 (2.3%). The graft was positioned flush to the anterior glenoid rim in the axial view in 34 patients (40.5%), medial in 34 (40.5%) and lateral in 16 (19%). If the “acceptable zone” of bone block placement was considered between 2 mm lateral and 4 mm medial to the glenoid rim, a too lateral position of the graft was found in seven patients (8.3%) and too medial position in ten (11.9%)—Table 7. The graft height evaluated in the sagittal plane was between 1 and 3 o’clock in 11 patients (13.1%), 2 and 4 o’clock in 25 (29.8%) and 3 and 5 o’clock in 45 (53.6%)—Table 8. The mean screw angle was 14° (SD 9)—Table 9. Screw protrusion into infraspinatus fossa was mean 6.2 mm (SD 4.6) for the superior and 4.7 mm (SD 3.7) for the inferior one. Postoperative hardware problems were reported in 13 (15.2%) patients—Table 10. Subscapularis muscle grade I infiltration was found in 14 patients (16.5%). As mentioned above, postoperative fractures of two grafts and three screws were correlated with recurrence (*p* = 0.0). All other parameters reported above on CT evaluation had no correlation with clinical results.

**Table 6:** Graft healing

	Number of patients
Graft healing	81 (95.3%)
Total graft lysis	1 (1.2%)
Superior screw—graft lysis	55 (64.7%)
Inferior screw—graft lysis	2 (2.3%)
Graft pseudoarthrosis	2 (2.3%)
Graft fracture	2 (2.3%)

In brackets % values

**Table 7:** Graft position—medial to lateral position in the axial plane

Graft position	Number of patients
Flush	34 (40.5%)
Medial	34 (40.5%)
Lateral	16 (19%)
Medial > 4 mm	10 (11.9%)
Lateral > 2 mm	7 (8.3%)

In brackets % values

**Table 8:** Graft position—height of graft in the sagittal plane using the o’clock description

Glenoid zones	Number of patients
1–3	11 (13.1%)
2–4	25 (29.8%)
3–5	45 (53.6%)
4–6	3 (3.6%)

In brackets % values

**Table 9:** Screw fixation

	Mean value	Median	Range values	SD
Superior screw angle (°)	14.1	12.5	0–42.4	9.0
Inferior screw angle (°)	14.2	12.6	0–40	9.1
Superior screw-equator angle (°)	17.6	16.7	0–41	7.8
Inferior screw-equator angle (°)	17.5	16.7	0–41	8.0
Superior screw protrusion (mm)	6.2	6	0-17.5	4.6
Inferior screw protrusion (mm)	4.7	5	0–14	3.7

In brackets % values

*SD* standard deviation

**Table 10:** Postoperative hardware problems

	Number of patients
Screw fractures	3 (3.5%)
Superior screw loosening	7 (8.2%)
Inferior screw loosening	2 (2.3%)
Both screws loosening	1 (1.2%)

In brackets % values

## Discussion

The most important finding of the present study was that intraoperative graft-related complications: fracture and the inability to achieve solid two-screw fixation were strong risk factors for recurrence. To our knowledge this is the first study to emphasise this fact.

This finding seems crucial as graft-related complications whilst harvesting, drilling and screw fixation were the most frequent problems in this study: seven out of eight cases. This could lead to the conclusion that if a surgeon encounters graft-related complications, conversion to open technique or changing the system of fixation (such as to the “button” system) might be considered [36, 37]. If this technical modification is able to improve results in these difficult cases requires further research. Other important findings were two factors influencing the final outcomes the most: SRSA and loss of external rotation with the arm at the side. Many patients reported “apprehension” to return to pre-injury sport activity. This situation was called “subjective return to sport anxiety”—it means patients were perfectly stable during clinical examination and daily activity, however, were afraid of getting back into overhead sports. SRSA was found in 41 patients (46.5%) and strongly influenced the clinical score results—it was qualified by the evaluating physician as the presence of “apprehension” in clinical scores, however, without any objective findings. In our previous studies, the term “subjective apprehension” was used; however, the term “apprehension” could be misleading, suggesting a poor outcome, which is not the case [19, 20]. Some hypotheses to explain the presence of SRSA were already presented including laxity, proprioceptive deficit after capsule excision and psychological effect; however, its origin and influence on long-term results remains unclear [38–42]. Flexion, abduction and loss of external rotation influenced the outcomes; however, the last factor seemed to have the strongest impact on clinical results. The mean loss of external rotation in this study was 15°, remaining comparable to data reported by the other authors [8, 12]. Ladermann et al. found it was related to the inside-out technique of the switching stick insertion from the posterior portal to determine the level of subscapularis split [43]. Using this technique, the split is performed higher than the recommended junction of the middle and inferior third of the muscle that could lead to positioning the graft too high—its consequence may be the increased tension of the conjoint tendon and loss of external rotation. Another reason might be capsule excision and a more aggressive subscapularis muscle split—in the arthroscopic technique a radiofrequency probe is used, not a gentle blunt splitting as in the open technique—responsible for scar formation. This could be a probable reason for loss of external rotation, as no correlation was found between the subscapularis muscle fatty infiltration and loss of external rotation.

Further identified limitations, however, without influence on clinical results were osteolysis of the superior part of the graft and imperfections in graft positioning in CT evaluation. Despite a very high graft fusion rate (81 patients—95.3%), graft osteolysis around the superior screw was found in 55 patients (64.7%). Zhu et al. found osteolysis in the superior part of the graft in 78.8% of the patients [16]. The other authors also reported this finding; however, it might be considered as a natural glenoid remodelling process [18, 44]. Bone block position in the sagittal plane (graft height) remains controversial: some authors recommend positioning the graft below the equator (3 o’clock), others believe the optimal position is between 2:30 to 4:20 or 2 and 5 o’clock [7, 11, 17]. In this study, the graft was below the equator in 48 patients (57.2%). If the proper graft position were judged between 2 and 5 o’clock, it would be reported in 70 patients (83.4%). However, there was still a visible number of grafts placed too high (11 patients—13.1%). In addition, Neyton et al. reported less precise graft positioning when compared to the open technique [45].

Hardware problems remained a further limitation reported in 13 patients (15.2%) in this study. It is important to note that screw problems were found in eight out of nine cases of revision after a primary arthroscopic Latarjet stabilisation. These findings are comparable to other reports [10, 12, 21, 46]. Shah et al. reported using cannulated screws as a risk factor [47]. Willemot et al. and Schmiddem et al. confirmed that monocortical fixation combined with cannulated screws provides less stable fixation compared to solid screws [48, 49]. It is possible to conclude that the use of a cannulated screw combined with any technical error could lead to complications like screw fracture or recurrence. The above findings confirmed the hypothesis that outcome analysis in this important cohort of patients would help to identify some “limitations” influencing the results of the arthroscopic Latarjet stabilisation technique. It is, however, crucial to remember that the methodology of this study is not free of certain limitations. Short-term follow-up is an important factor before any definitive conclusions are made; however, Griesser et al. reported that 73% of the recurrence occurred within the first 12 months after surgery [50]. In addition, Kee et al. found no important progression in graft osteolysis between 7.7 and 31.7 months of CT evaluation [44]. Preoperative radiographic parameters were not collected in a systematic manner so we decided not to include it in the study—this is why preoperative bone loss was not assessed in patient data. The clinical results of patients did not include data on postoperative pain, recovery and rehabilitation time to restore full activity, which are important factors in technique evaluation. Experience and technical skills of the surgeon could also strongly influence results. This study concerns the first patients operated on in 2011 as well as patients operated on almost 5 years later. This could be an important limitation of this study; however, it is the “natural history” of the implementation of a new technique. The strength of this study is based on a homogenous, single surgeon, large cohort of patients evaluated with both clinical examination and CT and follow-up rate exceeding 95%. Some clinical implications could be proposed. The implementation of the arthroscopic technique requires very meticulous training and preparation as intraoperative graft-related complications and lack of proper fixation are risk factors for recurrence. In case of these problems arising, the surgeon should be ready for a “salvage” procedure—conversion to open technique or changing the system of fixation. Hardware problems may be prevented by a gentle surgical technique and preoperative screw length planning [51]. The subscapularis muscle split level should be determined from “outside” like in the open technique and splitting should be performed gently, as scar tissue could be responsible for loss of external rotation with arm at the side.

## Conclusions

The arthroscopic Latarjet stabilisation procedure demonstrates satisfactory results upon clinical and radiographic evaluation in short-term follow-up; however, some factors influencing the outcomes were found. Intraoperative graft-related complications are a risk factor for recurrence. “Subjective return to sport anxiety” and loss of external rotation with the arm at the side are important factors worsening the results. Graft position imperfections and osteolysis of the superior part of the graft reported in CT evaluation do not influence the clinical results.
